# Optimising dual-energy CT scan parameters for virtual non-calcium imaging of the bone marrow: a phantom study

**DOI:** 10.1186/s41747-019-0125-2

**Published:** 2019-12-04

**Authors:** Felix C. Müller, Henrik Børgesen, Kasper Gosvig, Anders Rodell, Christian Booz, Bernhard Schmidt, Bernhard Krauss, Mikael Boesen

**Affiliations:** 1 Department of Radiology, Herlev and Gentofte Hospital, Herlev, Denmark; 2 Siemens Healthineers, Ballerup, Denmark; 3 Division of Experimental and Translational Imaging, Department of Diagnostic and Interventional Radiology, University Hospital Frankfurt, Frankfurt, Germany; 4 Siemens Healthineers, Forchheim, Germany; 5 Department of Radiology, Bispebjerg and Frederiksberg Hospital, Frederiksberg, Denmark

**Keywords:** Bone and bones, Bone marrow, Phantoms (imaging), Tomography (x-ray computed), MeSH does not recognize this term.

## Abstract

**Background:**

We investigated the influence of dose, spectral separation, pitch, rotation time, and reconstruction kernel on accuracy and image noise of virtual non-calcium images using a bone marrow phantom.

**Methods:**

The phantom was developed at our institution and scanned using a third-generation dual-source dual-energy CT scanner at five different spectral separations by varying the tube-voltage combinations (70 kV/Sn150 kV, 80 kV/Sn150 kV, 90 kV/Sn150 kV, and 100 kV/Sn150 kV, all with 0.6-mm tin filter [Sn]; 80 kV/140 kV without tin filter) at six different doses (volume computed tomography dose index from 1 to 80 mGy). In separate experiments, rotation times, pitch, and reconstruction kernels were varied at a constant dose and tube voltage. Accuracy was determined by measuring the mean error between virtual non-calcium values in the fluid within and outside of the bone. Image noise was defined as the standard deviation of virtual non-calcium values.

**Results:**

Spectral separation, dose, rotation time, or pitch did not significantly correlate (*p* > 0.083) with mean error. Increased spectral separation (*r*_s_-0.96, *p* < 0.001) and increased dose (*r*_s_-0.98, *p* < 0.001) correlated significantly with decreased image noise. Increasing sharpness of the reconstruction kernel correlated with mean error (*r*_s_ 0.83, *p* = 0.015) and image noise (*r*_s_ 1.0, *p* < 0.001).

**Conclusions:**

Increased dose and increased spectral separation significantly lowered image noise in virtual non-calcium images but did not affect the accuracy. Virtual non-calcium reconstructions with similar accuracy and image noise could be achieved at a lower tube-voltage difference by increasing the dose.

## Keypoints

- Increasing dose or spectral separation reduced image noise in virtual non-calcium images
- Accuracy of virtual non-calcium images is unaffected by dose
- High spectral separation can reduce the dose at equal image noise in virtual non-calcium images

## Background

A dual-energy computed tomography (DECT) scan can be created by combining two datasets acquired over the same region with different x-ray spectra (low kV and high kV) [1]. Virtual non-calcium (VNCa) images are created from DECT data by estimating the amount of bone mineral, primarily made up of calcium, and subtracting this from the original image. If this is done on the cancellous bone, then the resulting VNCa image will display the bone marrow underlying the bone mineral.

VNCa images can characterise bone marrow changes associated with fractures, multiple myeloma and rheumatoid arthritis [2–4]. A number of studies have demonstrated that VNCa can demonstrate bone marrow oedema associated with acute fractures [2, 5–8]. However, those studies used different DECT scan parameters, including tube-voltage combinations and doses, and virtual phantom studies have demonstrated that tube-voltage combinations and dose affect image noise in DECT scans [9]. But the interaction between tube-voltage combinations and dose for the characterisation of the bone marrow by DECT has not yet been investigated, neither *in vivo* nor in physical phantoms. Therefore, while we know that tube-voltage combination and dose do affect image noise in VNCa images, we cannot easily compare the results between scans or studies, which have used different DECT scan parameters.

In DECT, the difference between the two x-ray spectra is referred to as the spectral separation. One approach to increase the spectral separation is through the use of tin filters in front of the x-ray tube or by choosing two tube voltages which are further apart [10]. VNCa images are calculated from the DECT datasets by a three-material decomposition, where the original data is decomposed into bone mineral, yellow marrow and red marrow, [2]. The dual-energy ratio (DE_ratio_) for bone mineral must be defined before the decomposition and can be experimentally determined by dividing the slope of the attenuation of different concentrations of the bone mineral obtained at low kV and high kV [11]. The red marrow and yellow marrow are defined by their respective attenuation on the low kV and high kV image, expressed in Hounsfield units (HU) [10].

Computed tomography (CT) parameters, which directly affect the noise in one of the images, also affect the noise in VNCa images [12]. In addition, higher spectral separation results in a higher DE_ratio_ and a more precise calculation of the bone mineral content [9, 13, 14]. Therefore, a higher DE_ratio_ should result in a less noisy VNCa image. Inversely, the degree of spectral separation can be inferred, when the DE_ratio_ is known [12]_._

The accuracy of a VNCa image can be estimated by measuring the difference between VNCa values and true non-calcium values in the bone marrow. In an idealised phantom, where VNCa values are measured in a bone marrow analogue both without bone and with bone, the mean difference between the two measurements should be zero, if the test is completely accurate.

We hypothesised that an increased spectral separation, higher dose, lower pitch, longer rotation time, and softer reconstruction kernel are correlated with decreased image noise, but do not affect accuracy in VNCa images. We tested this hypothesis prospectively using an idealised phantom of different bone marrow compositions.

## Methods

No institutional review board approval was necessary for this prospective phantom study.

### Phantom design

**Fig. 1:**
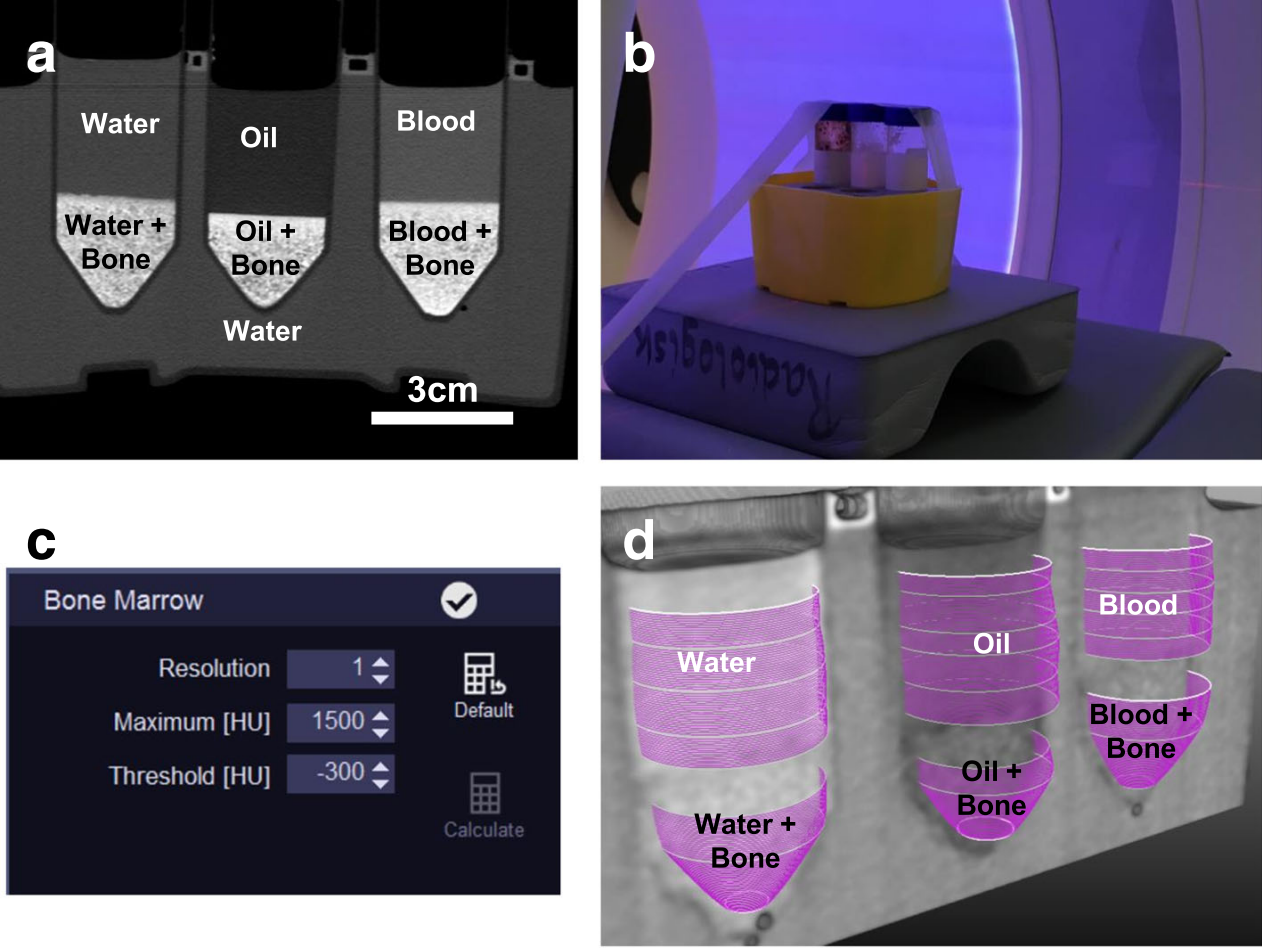
Bone marrow phantom for dual-energy CT scanners. **a** Reformat of an ultra-high-resolution CT scan of three tubes filled with water, oil (as yellow marrow analogue/YMA) and blood (as red marrow analogue/RMA) and partially filled with bone granulate. Note that the granulate was more densely packed in the transition between fluid with bone and fluid without bone. This area was avoided in the analysis. **b** The phantom as placed in the CT scanner. **c** Settings used in the bone marrow oedema application class in Syngo.Via. **d** 3D rendering of the volumes of interest measured, which gives six unique measurement compartments, three compartments of fluid with bone and three of fluid without bone

A custom idealised phantom was developed at our institution by filling three polyvinyl chloride tubes (diameter 27 mm) with approximately 20 mL of one of the three fluids: red marrow analogue (RMA), yellow marrow analogue (YMA), or water. Each tube was also partially filled with 7.5 mL of bone granulate (Fig. 1a). This resulted in six compartments: three compartments of fluid without bone and three compartments of fluid with bone.

For RMA, heparinised human full blood was used, while for YMA, sunflower seed oil was used. Bone granulate was extracted from the cancellous bone from the neck of the femur in young cows. The bone was rinsed and dried before being crushed using a coarse grinder. All tubes were arranged in line and submerged in a 12 × 7 cm water chamber. All six compartments were placed equidistant from the isocentre of the scanner (Fig. 1b), and the entire phantom fixed to the scan table.

### Dual-energy scan series

Scans were performed on a third-generation dual-source dual-energy system (Somatom Force, Siemens Healthineers, Forchheim, Germany). All scans were performed in a single session in August 2018. Doses for each scan were estimated during the setup of the scan protocol at the scanner and expressed as the volume computed tomography dose index (CTDI_vol_).

In the first experiment used for calibration, the phantom was scanned at a constant CTDI_vol_ of 80 mGy at all five available tube-voltage combinations (70 kV/Sn150 kV, 80 kV/Sn150 kV, 90 kV/Sn150 kV, 100 kV/Sn150 kV, and 80 kV/140 kV; Sn indicating the use of a 0.6-mm tin filter). In addition, a single scan was performed in a high-resolution mode. In the second experiment, the phantom was again scanned with all five available tube-voltage combinations. For each tube-voltage combination, the phantom was scanned with six tube currents adjusted to result in equal doses. The CTDI_vol_ for the six scans was between 1 and 80 mGy. In the third experiment, the tube-voltage combination was kept constant, but the rotation time was varied between 0.25 s and 1 s. To keep a constant CTDI_vol_, the tube current was reduced correspondingly. In the fourth experiment, the pitch was varied between 0.3 and 1.2 with constant CTDI_vol_ and a constant tube-voltage combination. In the fifth experiment, the phantom was scanned once but images were reconstructed with eight different quantitative kernels with increasing sharpness. The automatic tube current modulation was disabled for all scans. Exact scan and postprocessing parameters are listed in Additional file 1: Table S1.

### Image postprocessing

CT series were postprocessed on a commercially available three-dimensional workstation (syngo.via version VB20-HF5, Siemens Healthineers, Forchheim, Germany) using the bone marrow oedema algorithm. Results from the first experiment were used to calibrate the settings of the three-material decomposition, by a single investigator. For each tube-voltage combination, scanned at CTDI_vol_ of 80 mGy, mean HU values of YMA and RMA without bone were measured on both the high and low kV series. The DE_ratio_ (referred to as ‘rel. Calcium’ in syngo.via) was then increased stepwise, and for each step, a three-material decomposition was done. For each DE_ratio_, the mean difference between the fluid with bone and without bone for each of the three fluids was calculated, by measuring the mean CT numbers on the calculated VNCa image. The DE_ratio_ which yielded the lowest mean error was then chosen as the base for three-material decomposition in the rest of the experiments. Other syngo.via specific settings in the bone marrow oedema application were set to resolution 1, maximum 1500 HU and threshold -300 HU.

### Measurement of VNCa numbers

The VNCa numbers were measured using a custom network in MeVisLab (version 2.8.2, MeVisLab GmbH, Bremen, Germany) by a single investigator. Using the high-resolution reconstruction, six volumes of interest (VOIs) were placed; one in each of the six compartments. Each VOI was placed so that it contained as much of the compartment as possible, while avoiding entrapped air and the irregular border between the fluid with bone and without bone. VOIs contained 221,603, 235,183, and 189,754 voxels in the three fluid compartments without bone and 113,279, 81,423, and 116,106 voxels in the three fluid compartments with bone.

VOIs at identical positions were automatically transferred and reused for all scan series. Mean HU values in the VNCa images in each of the six compartments were automatically measured within MeVisLab. Image noise was defined as the mean standard deviation of the VNCa measurements in the three compartments containing fluid and bone. Accuracy was defined as the mean error between fluid with and without bone as outlined in Eq. 1.

1 $$ \mathrm{Mean}\ \mathrm{error}=\frac{\mid {\mathrm{YMA}}_{\mathrm{bone}}-{\mathrm{YMA}}_{\mathrm{w}/\mathrm{o}\mathrm{bone}}\mid +\mid {\mathrm{RMA}}_{\mathrm{bone}}-{\mathrm{RMA}}_{\mathrm{w}/\mathrm{o}\mathrm{bone}}\mid +\mid {\mathrm{Water}}_{\mathrm{bone}}-{\mathrm{Water}}_{\mathrm{w}/\mathrm{o}\mathrm{bone}}\mid }{3} $$

### Statistics

Data was analysed using freely available statistical software R (version 3.4.1; R foundation, Vienna, Austria). The correlation between image noise and pitch, rotation time, sharpness of the reconstruction kernel, spectral separation and dose was calculated using the Spearman’s rank correlation coefficient (*r*_s_). Since dose and spectral separation were measured multiple times, they were normalised so that when determining the correlation between dose and image noise and mean error, the tube-voltage combination of 70 kV/Sn150 kV was defined as 1, while for spectral separation, the image noise and mean error at 80 mGy was defined as 1. Since the three-material decomposition was based on a custom calibration, which minimised the mean error for each tube-voltage combination individually, the influence of the spectral separation on the mean error could not be determined in this experiment. A *p* value below 0.05 was considered to indicate a significant correlation, with no correction applied for multiple testing.

## Results

All results are also listed in numerical form in Additional file 1: Table S2.

### Calibration and determination of spectral separation

The lowest achievable mean error was between 10.5 and 14.0 HU for the five different tube-voltage combinations. DE_ratios_ were higher in all tube-voltage combinations which used a tin filter with the highest DE_ratio_ at 70 kV/Sn150 kV and the lowest at 80 kV/140 kV. The calibration values which resulted in the lowest mean error for this phantom are listed in Table 1.

**Table 1:** Calibration of virtual non-calcium images and measurement of spectral separation

Tube-voltage combination	Yellow marrow low kV (HU)	Yellow marrow high kV (HU)	Red marrow low kV (HU)	Red marrow high kV (HU)	DE_ratio_
70 kV/Sn150 kV	-130	-81	81	61	2.27
80 kV/Sn150 kV	-115	-80	80	60	1.99
90 kV/Sn150 kV	-115	-82	75	60	1.81
100 kV/Sn150 kV	-105	-81	72	61	1.67
80 kV/140 kV	-130	-98	75	70	1.47

For each tube-voltage combination, the Hounsfield units for the yellow marrow and red marrow were measured. The dual-energy ratio which yielded the lowest mean error was then determined empirically and is used as a measure of spectral separation. Spectral separation increases when the difference in tube voltage is larger or when the tin filter is used; *DE*_*ratio*_ dual-energy ratio; *HU* Hounsfield units; *Sn* indicates the use of a 0.6-mm tin filter

### Spectral separation and dose

An increase of dose at constant spectral separation was significantly inversely correlated to image noise (*r*_s_-0.98, *p* < 0.001) while the mean error remained unchanged (*r*_s_ 0.17, *p* = 0.365). In addition, an increase in spectral separation was significantly inversely correlated to image noise (*r*_s_-0.96, *p* < 0.001) for all doses.

**Fig. 2:**
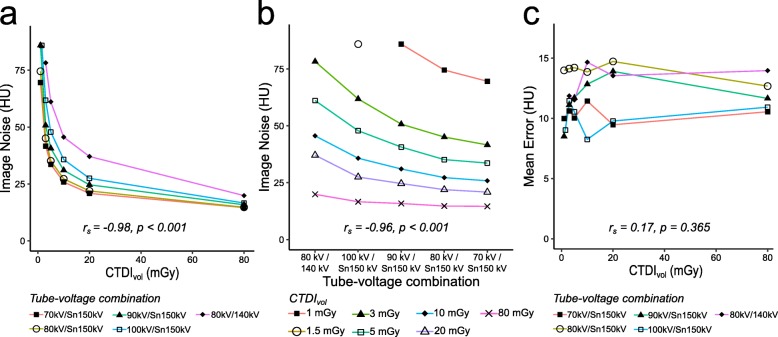
Increased spectral separation and dose decrease noise in virtual non-calcium images. Image noise decreases continuously as dose (**a**) and spectral separation (**b**) is increased. Image noise is defined as the standard deviation in the bone on virtual non-calcium images. Tube-voltage combinations are sorted with increasing spectral separation from left to right in (**b**). CTDI_vol_ could not be set below 1.5 mGy at 100 kV/Sn150 kV and 3 mGy at 80 kV/140 kV. **c** No correlation can be seen between increased dose and accuracy, defined as mean error between the fluid within bone against the same fluid outside bone. CTDI_vol_ volume computed tomography dose index; HU Hounsfield units; *r*_s_ Spearman rank correlation coefficient; *Sn* indicates the use of a 0.6-mm tin filter

**Fig. 3:**
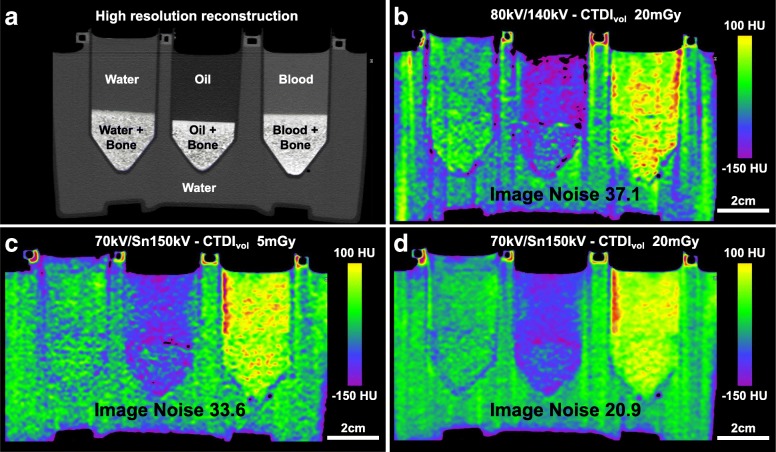
Higher spectral separation can compensate for the increase in image noise in lower dose virtual non-calcium images. High-resolution conventional reconstruction (**a**) and virtual non-calcium reconstructions (**b−d**) at three different tube-voltage and dose combinations at the same position. Image noise for each reconstruction is stated and is the mean standard deviation for the three chambers with fluid and bone. Notice in **b** and **d** a volume computed tomography dose index of 20 mGy, but the higher difference in tube voltage and use of a tin filter leads to a reduction in image noise. While at a constant tube-voltage combination in **c** and **d**, the increase in dose leads to a decrease in image noise. Similar image noise is seen at a four times reduced dose between **b** and **c**, indicating that scans with lower spectral separation must increase the dose to maintain similar image noise; *CTDI*_*vol*_ Volume computed tomography dose index; *Sn* indicates the use of a tin filter

Figure 2 demonstrates the relationship between dose and spectral separation and shows that the image noise depends on both the dose and tube-voltage combination. Increasing the spectral separation allowed for a decrease in dose at comparable image noise. This is demonstrated in Fig. 3, where a similar image can be achieved with four times less dose, while when scanned at the same dose the image noise is visibly reduced.

### Pitch, rotation time and reconstruction kernel

**Fig. 4:**
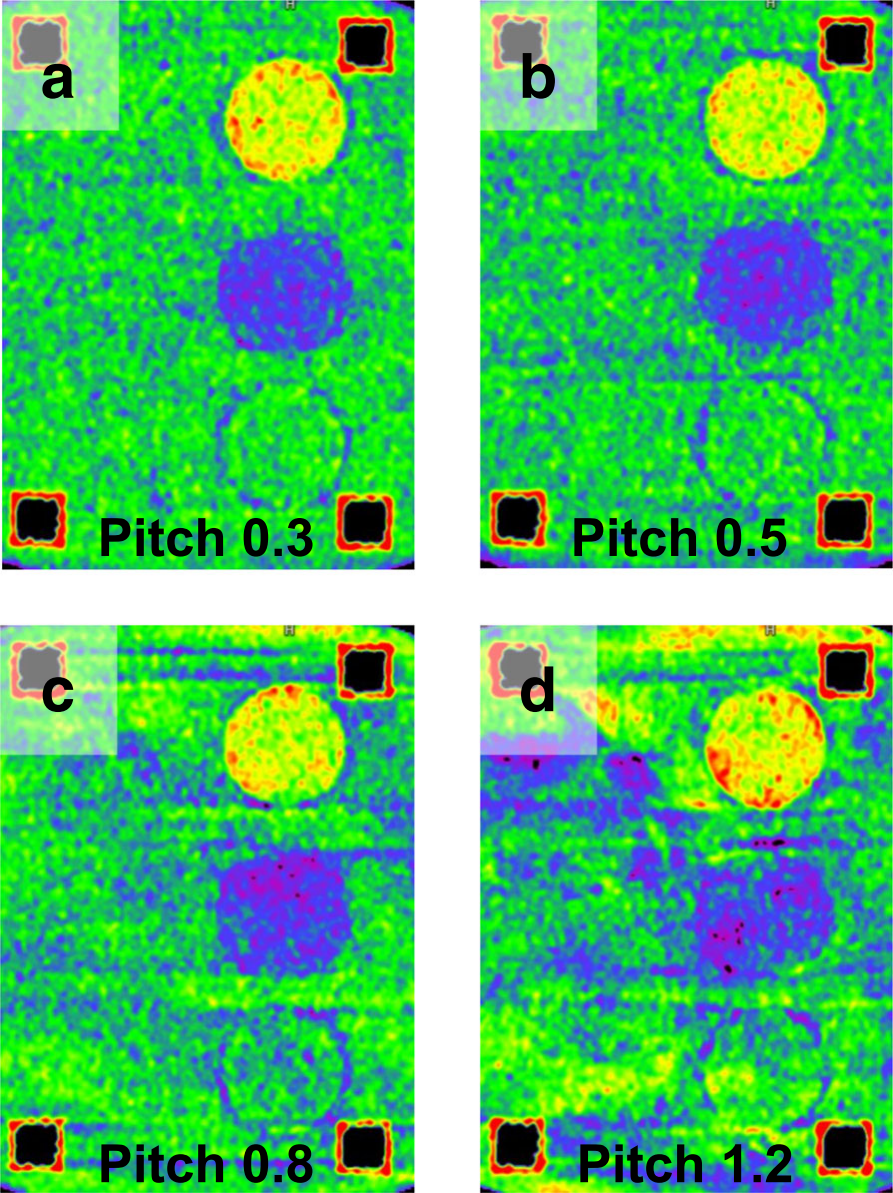
Lower pitch image acquisition reduced spiral-like artefacts in virtual non-calcium images. Orthogonal reformats (**a–d**) of virtual non-calcium images acquired at different pitch angles. Notice that spiral-like artefacts clearly visible at pitch 1.2 and 0.8 have almost disappeared at pitch 0.3

An increase in pitch at constant CTDI_vol_ was not significantly correlated to image noise (*r*_s_ 1.0, *p* = 0.083) or mean error (*r*_s_-0.60, *p* = 0.417). However, lowering the pitch seemed to reduce spiralling artefacts which could be seen in the higher pitch VNCa reconstructions, with the best results at a pitch of 0.3. (Fig. 4).

Rotation time did not significantly correlate with image noise (*r*_s_-0.80, *p* = 0.333) or mean error (*r*_s_-0.80, *p* = 0.333).

**Fig. 5:**
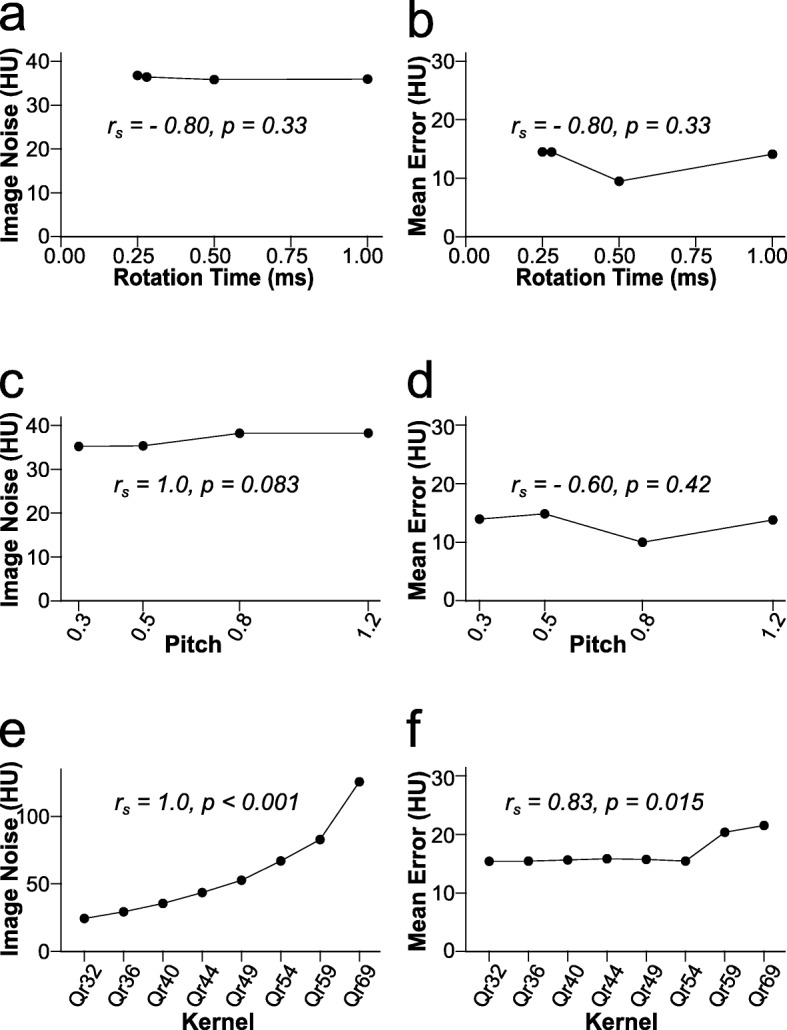
Influence of rotation time, pitch and reconstruction kernel on accuracy and image noise. Shown is the influence of rotation time, pitch, and the reconstruction kernel on image noise (**a**, **c**, **e**) and accuracy (**b**, **d**, **f**) of virtual non-calcium images. Only the reconstruction kernel significantly correlated with accuracy and image noise. Reconstruction kernels (Qr32 through Qr69) in (**e**, **f**) are sorted with increasing kernel sharpness from left to right; *HU* Hounsfield units; *r*_s_ Spearman rank correlation coefficient

An increase in the sharpness of the reconstruction kernel was significantly correlated with image noise (*r*_s_ 1.0, *p* < 0.001) and mean error (*r*_s_ 0.83, *p* = 0.015). This increase in mean error occurred with the two sharpest kernels (Qr59 and Qr69) only. Mean error for the other six kernels ranged between 15.4 and 15.8 HU, and additional analysis found no significant correlation between those six kernels and mean error (*r*_s_ 0.6, *p* = 0.242) (Fig. 5).

## Discussion

This phantom study found that increased dose and spectral separation was significantly correlated with lower image noise, while sharper reconstruction kernels were significantly correlated with increased image noise. The lowest image noise was achieved at a tube-voltage combination of 70 kV/Sn150 kV for all measured doses. Rotation time and pitch were not found to be significantly correlated with image noise, but visual inspections of lower pitch images showed reduced spiralling artefacts. The accuracy, measured as the mean error between fluid with and without bone, was not significantly correlated to any DECT scan parameter, but affected by very sharp reconstruction kernels. Our study is in agreement with previous studies demonstrating that a dose saving can be achieved when increasing the spectral separation through the use of a tin filter [11, 12]. Results are also in agreement with results from previous phantom studies [15, 16] addressing the capability of different scanners to quantify iodine concentrations. In our study, the lowest spectral separation at the lowest dose had the same accuracy as all other scan series. This indicates that the measurement of the accuracy of calcium quantification alone is not a valuable parameter for judging the quality of a DECT scan, but should be supplemented by the measure of image noise or spatial resolution. Results are likely similar in virtual non-contrast images.

While the highest CTDI_vol_ of 80 mGy resulted in the lowest image noise, the ideal combination of low dose and low image noise seemed to be between 5 and 10 mGy.

Considering the results of this study for clinical imaging of bone marrow with DECT, it would suggest this best be done at a high spectral separation, ideally at a tube-voltage combination of 70 kV/Sn150 kV and a CTDI_vol_ between 5 and 10 mGy. When a lower spectral separation is chosen, a corresponding dose increase would be necessary to maintain a similar image noise. This is likely the case in the spine and pelvic exams, where imaging at a tube voltage 70 kV is not feasible.

There are limitations to this study which warrant further discussion. Firstly, only four unique scans were available for rotation time and pitch investigations. An increased number of scans might have demonstrated a significant correlation between pitch and image noise. However, the image noise in the VNCa images fell from 38.2 to 35.2 HU when reducing pitch and from 36.8 to 35.9HU for increased rotation time—so the effect of pitch and rotation time on image noise would be small in our opinion. Secondly, the phantom is not a direct representation of a human bone. The phantom does not have a representation of dense cortical bone, which can result in beam hardening and more noisy VNCa images. In addition, the size of the phantom corresponds roughly to a human wrist or ankle and is very different from the size of the abdomen imaged in a spinal or pelvic CT examination. However, the underlying principle of correlation between spectral separation, dose and image noise still apply. Thirdly, an analogue material was used in the construction of the phantom. The bone granulate used in this study is more densely packed than the normal cancellous bone in humans and mechanically and chemically altered. Also, we used heparinised full blood as a red marrow analogue and unfiltered sunflower seed oil as a yellow marrow analogue. We found the measurement of our analogue material to correspond well to the default HU settings of red marrow, yellow marrow and the DE_ratio_ of calcium used in clinical practice. Fourthly, we only studied the bone marrow phantom, using three-material decomposition based on DECT scans from a dual-source scanner. While the same principles also apply to dual-energy scans based on sequential image acquisition or rapid kilovolt-switching, the effect of scan parameters on VNCa images when using dual-layer detectors might be different. Finally, we studied the effect of scan parameters on image quality in an idealised phantom. The clinical indication usually relates to the demonstration of bone marrow oedema, and therefore, the size of the oedema needs to be taken into account, with smaller oedemas in smaller bones better visualised with higher spatial resolution, *i.e*, lower image noise. As we demonstrated in this study, the use of a smooth reconstruction kernel does decrease the noise in VNCa images; this however decreases the spatial resolution of the VNCa image. We did not investigate the spatial resolution of the VNCa images in our phantom study; this should be taken into consideration when evaluating postprocessing techniques for the reduction of image noise.

In conclusion, we found that the accuracy of VNCa measurements in the bone was not affected by pitch, rotation time, spectral separation, or dose. The sharpest reconstruction kernels negatively affected the accuracy. Image noise was significantly correlated with spectral separation, dose, and reconstruction kernel. Visual inspection demonstrated a noticeable reduction of spiral-like artefacts in low pitch imaging. Extrapolating information from this phantom study to an optimal clinical DECT scan protocol for characterising the bone marrow suggests the need for high spectral separation ideal close to a tube-voltage combination of 70 kV/Sn150 kV, a dose between 5 and 10 mGy, a pitch of 0.3 and a reconstruction kernel as soft as clinically achievable. When using scanners not capable of this high spectral separation or in body regions where this is infeasible (*e.g*, pelvis or abdominal scans), the dose has to be increased to compensate for the increased image noise.

## Supplementary information

**Additional file 1: Table S1.** Scan parameters. **Table S2.** All experimental results.

## Data Availability

The datasets used and/or analysed during the current study are available from the corresponding author on reasonable request.

## Abbreviations

CT: Computed tomography
CTDI_vol_: Volume CT dose index
DECT: Dual-energy CT
DE_ratio_: Dual-energy ratio
HU: Hounsfield units
RMA: Red marrow analogue
*r*_s_: Spearman rank correlation coefficient
VNCa: Virtual non-calcium
VOI: Volume of interest
YMA: Yellow marrow analogue
