# Supplementary material for: Optimising dual-energy CT scan parameters for virtual non-calcium imaging of the bone marrow: a phantom study
Source: Eur Radiol Exp. 2019 Dec 4;3:46. doi: 10.1186/s41747-019-0125-2 (PMC6892987; doi:10.1186/s41747-019-0125-2)
Supplement: Supplementary file 1 — Additional file 1: Table S1. Scan parameters. Table S2. All experimental results. [file 41747_2019_125_MOESM1_ESM.docx]

# Table S1: Scan parameters

**High resolution reference scan**

| **Scan Series** | **Tube-voltage Combination (kV)** | **Rotation Time (s)** | **Pitch** | **Tube Current (mAs)** | **CTDI_Vol_** | **Collimation** |
| --- | --- | --- | --- | --- | --- | --- |
| 0_0^a)^ | 120 | 1,0 | 0,2 | 685 | 40,11 | 64 x 0,6 |

**Experiment 1 and 2: Calibration, Dose and Tube Voltage**

| **Scan Series** | **Tube-voltage Combination (kV)** | **Rotation Time (s)** | **Pitch** | **Tube Current (mAs)** | **CTDI_Vol_** | **Collimation** |
| --- | --- | --- | --- | --- | --- | --- |
| 1_1 | 70 / Sn150 | 0,5 | 0,7 | 44 | 1,02 | 128 x 0,6 |
| 1_2 | 70 / Sn150 | 0,5 | 0,7 | 131 | 3,02 | 128 x 0,6 |
| 1_3 | 70 / Sn150 | 0,5 | 0,7 | 218 | 5,01 | 128 x 0,6 |
| 1_4 | 70 / Sn150 | 0,5 | 0,7 | 473 | 10,00 | 128 x 0,6 |
| 1_5 | 70 / Sn150 | 0,5 | 0,7 | 875 | 20,00 | 128 x 0,6 |
| 1_6 ^d)^ | 70 / Sn150 | 1,0 | 0,3 | 3500 | 79,93 | 128 x 0,6 |

| **Scan Series** | **Tube-voltage Combination (kV)** | **Rotation Time (s)** | **Pitch** | **Tube Current (mAs)** | **CTDI_Vol_** | **Collimation** |
| --- | --- | --- | --- | --- | --- | --- |
| 2_1 | 80 / Sn150 | 0,5 | 0,7 | 24 | 1,01 | 128 x 0,6 |
| 2_2 | 80 / Sn150 | 0,5 | 0,7 | 73 | 3,04 | 128 x 0,6 |
| 2_3 | 80 / Sn150 | 0,5 | 0,7 | 121 | 5,02 | 128 x 0,6 |
| 2_4 | 80 / Sn150 | 0,5 | 0,7 | 243 | 10,03 | 128 x 0,6 |
| 2_5 | 80 / Sn150 | 0,5 | 0,7 | 486 | 20,00 | 128 x 0,6 |
| 2_6 ^d)^ | 80 / Sn150 | 1,0 | 0,3 | 1945 | 80,16 | 128 x 0,6 |

| **Scan Series** | **Tube-voltage Combination (kV)** | **Rotation Time (s)** | **Pitch** | **Tube Current (mAs)** | **CTDI_Vol_** | **Collimation** |
| --- | --- | --- | --- | --- | --- | --- |
| 3_1 | 90 / Sn150 | 0,5 | 0,7 | 16 | 1,02 | 128 x 0,6 |
| 3_2 | 90 / Sn150 | 0,5 | 0,7 | 48 | 3,00 | 128 x 0,6 |
| 3_3 | 90 / Sn150 | 0,5 | 0,7 | 81 | 5,04 | 128 x 0,6 |
| 3_4 | 90 / Sn150 | 0,5 | 0,7 | 162 | 10,03 | 128 x 0,6 |
| 3_5 | 90 / Sn150 | 0,5 | 0,7 | 323 | 20,00 | 128 x 0,6 |
| 3_6 ^d)^ | 90 / Sn150 | 1,0 | 0,3 | 1295 | 80,12 | 128 x 0,6 |

| **Scan Series** | **Tube-voltage Combination (kV)** | **Rotation Time (s)** | **Pitch** | **Tube Current (mAs)** | **CTDI_Vol_** | **Collimation** |
| --- | --- | --- | --- | --- | --- | --- |
| 4_1^b)^ | 100 / Sn150 | 0,5 | 0,7 | 24 | 1,50 | 128 x 0,6 |
| 4_2 | 100 / Sn150 | 0,5 | 0,7 | 48 | 3,01 | 128 x 0,6 |
| 4_3 | 100 / Sn150 | 0,5 | 0,7 | 80 | 4,97 | 128 x 0,6 |
| 4_4 | 100 / Sn150 | 0,5 | 0,7 | 161 | 10,05 | 128 x 0,6 |
| 4_5 | 100 / Sn150 | 0,5 | 0,7 | 321 | 20,00 | 128 x 0,6 |
| 4_6 ^d)^ | 100 / Sn150 | 1,0 | 0,3 | 1290 | 80,12 | 128 x 0,6 |

| **Scan Series** | **Tube-voltage Combination (kV)** | **Rotation Time (s)** | **Pitch** | **Tube Current (mAs)** | **CTDI_Vol_** | **Collimation** |
| --- | --- | --- | --- | --- | --- | --- |
| 5_-^b)^ | - | - | - | - | - | - |
| 5_2 | 80 / 140 | 0,5 | 0,7 | 66 | 3,07 | 128 x 0,6 |
| 5_3 | 80 / 140 | 0,5 | 0,7 | 110 | 5,00 | 128 x 0,6 |
| 5_4 | 80 / 140 | 0,5 | 0,7 | 223 | 10,00 | 128 x 0,6 |
| 5_5 | 80 / 140 | 0,5 | 0,7 | 445 | 20,03 | 128 x 0,6 |
| 5_6 ^d)^ | 80 / 140 | 1,0 | 0,3 | 1780 | 80,00 | 128 x 0,6 |

**Experiment 3: RotationTine**

| **Scan Series** | **Tube-voltage Combination (kV)** | **Rotation Time (s)** | **Pitch** | **Tube Current (mAs)** | **CTDI_Vol_** | **Collimation** |
| --- | --- | --- | --- | --- | --- | --- |
| 6_1 | 80 / Sn150 | 0,25 | 0,7 | 121 | 5,00 | 128 x 0,6 |
| 6_2 | 80 / Sn150 | 0,28 | 0,7 | 121 | 5,00 | 128 x 0,6 |
| 6_3 | 80 / Sn150 | 0,5 | 0,7 | 121 | 5,02 | 128 x 0,6 |
| 6_4 | 80 / Sn150 | 1,0 | 0,7 | 121 | 5,02 | 128 x 0,6 |

**Experiment 4: Pitch**

| **Scan Series** | **Tube-voltage Combination (kV)** | **Rotation Time (sec)** | **Pitch** | **Tube Current (mAs)** | **CTDI_Vol_** | **Collimation** |
| --- | --- | --- | --- | --- | --- | --- |
| 7_1 | 80 / Sn150 | 0,5 | 0,3 | 121 | 5,11 | 128 x 0,6 |
| 7_2 | 80 / Sn150 | 0,5 | 0,5 | 121 | 5,03 | 128 x 0,6 |
| 7_3 | 80 / Sn150 | 0,5 | 0,8 | 121 | 5,00 | 128 x 0,6 |
| 7_4 | 80 / Sn150 | 0,5 | 1,2 | 121 | 5,02 | 128 x 0,6 |

**Experiment 5: Reconstruction Kernel**

| **Scan Series** | **Tube-voltage Combination (kV)** | **Rotation Time (s)** | **Pitch** | **Tube Current (mAs)** | **CTDI_vol_** | **Collimation** |
| --- | --- | --- | --- | --- | --- | --- |
| 8_1^c)^ | 80 / Sn150 | 0,5 | 0,7 | 121 | 5,02 | 1. 0,6 |

Scanned on Siemens Somatom Force on the 15.08.2018 at Herlev Hospital. Scans were reconstructed using kernel Qr40 with a slice thickness of 0.75 mm and a spacing of 0.5 mm a) Scanned using ultra high resolution scan mode b) at a Tube-voltage combination of 100 kV / Sn150 kV and 80 kV / 140 kV the scanner could not be reduced in CTDI_vol_ to 1 mGy c) Reconstructed using kernels Qr32 - Qr36 - Qr40 - Qr44 - Qr49 - Qr54 - Qr59 - Qr69 d) scans used for calibration of the three material decomposition;

*CTDIvol* volume computed tomography dose index; *Sn* Indicates use of 0.6mm tin filter;

# Table S2: All experimental results

| **S2a) Dose & Tube-voltage (image noise)** | | | | | | | | | | | | | | | | |  | | |  | |  | | |  |  |
| --- | --- | --- | --- | --- | --- | --- | --- | --- | --- | --- | --- | --- | --- | --- | --- | --- | --- | --- | --- | --- | --- | --- | --- | --- | --- | --- |
|  | ***Tube voltage combination*** | | | | | | | | | | | | | | | |  | | |  | |  | | |  |  |
| ***CTDI_Vol_ (mGy)*** | ***70 kV / Sn150 kV*** | | | ***80kV / Sn150 kV*** | | | ***90 kV / Sn150 kV*** | | | | ***100 kV / Sn150 kV*** | | | ***80 kV / 140 kV*** | | |  | | |  | |  | | |  |  |
| ***1*** | 69.6 | | | 74.5 | | | 85.9 | | | | 85.9 ^a)^ | | | - ^b)^ | | |  | | |  | |  | | |  |  |
| ***3*** | 41.6 | | | 45.1 | | | 50.7 | | | | 61.8 | | | 78.3 | | |  | | |  | |  | | |  |  |
| ***5*** | 33.6 | | | 35.1 | | | 40.7 | | | | 47.8 | | | 61.1 | | |  | | |  | |  | | |  |  |
| ***10*** | 25.8 | | | 27.2 | | | 31.0 | | | | 35.8 | | | 45.6 | | |  | | |  | |  | | |  |  |
| ***20*** | 20.9 | | | 21.9 | | | 24.6 | | | | 27.5 | | | 37.1 | | |  | | |  | |  | | |  |  |
| ***80*** | 14.6 | | | 14.7 | | | 15.9 | | | | 16.6 | | | 19.9 | | |  | | |  | |  | | |  |  |
| *Tube voltage combination* | | | | | | | *r_s_* -0.98 | | | | *p < 0.001* | | |  | | |  | | |  | |  | | |  |  |
| *Dose (CTDI_vol_)* | | | | | | | r_s_ -0.96 | | | | *p < 0.001* | | |  | | |  | | |  | |  | | |  |  |
|  | | | | | | | | | | | | | | | | |  | | |  | |  | | |  |  |
| **S2b) Dose & Tube-voltage (mean error)** | | | | | | | | | | | | | | | | |  | | |  | |  | | |  |  |
|  | ***Tube voltage combination*** | | | | | | | | | | | | | | | |  | | |  | |  | | |  |  |
| ***CTDI_Vol_ (mGy)*** | ***70kV/ Sn150kV*** | | | ***80kV/ Sn150kV*** | | | ***90kV/ Sn150kV*** | | | | ***100kV/ Sn150kV*** | | | ***80kV/ 140kV*** | | |  | | |  | |  | | |  |  |
| ***1*** | 10.0 | | | 14.0 | | | 8.5 | | | | 9.0 ^a)^ | | | - ^b)^ | | |  | | |  | |  | | |  |  |
| ***3*** | 10.6 | | | 14.1 | | | 11.1 | | | | 11.5 | | | 11.9 | | |  | | |  | |  | | |  |  |
| ***5*** | 10.0 | | | 14.2 | | | 11.7 | | | | 10.5 | | | 11.5 | | |  | | |  | |  | | |  |  |
| ***10*** | 11.4 | | | 13.9 | | | 12.8 | | | | 8.2 | | | 14.7 | | |  | | |  | |  | | |  |  |
| ***20*** | 9.5 | | | 14.7 | | | 13.9 | | | | 9.8 | | | 13.5 | | |  | | |  | |  | | |  |  |
| ***80*** | 10.5 | | | 12.7 | | | 11.7 | | | | 10.9 | | | 14.0 | | |  | | |  | |  | | |  |  |
| Dose (CTDI_vol_) | | | | | | | *r_s_ 0.17* | | | | p = 0.365 | | |  | | |  | | |  | |  | | |  |  |
|  | | | | | | | | | | | | | | | | |  | | |  | |  | | |  |  |
|  | | | | | | | | | | | | | | | | | | | | | | | |  |  |  |
| **S2c) Rotation Time** | | | | | | | | | | | | | | | | | | | | | | | |  |  |  |
|  | | | ***Rotation Time*** | | | | | | |  | | |  | |  | | | | | |  | | | |  |  |
|  | | | *0.25 s* | | *0.28 s* | *0.5 s* | | *1.0 s* | | ***r_s_*** | | | ***p*** | |  | | | | | |  | | | |  |  |
| ***Mean Error*** | | | 14.5 | | 14.5 | 9.5 | | 14.1 | | -0.80 | | | 0.333 | |  | | | | | |  | | | |  |  |
| ***Image Noise*** | | | 36.8 | | 36.4 | 35.8 | | 35.9 | | -0.80 | | | 0.333 | |  | | | | | |  | | | |  |  |
|  | | | | | | | | | | | | | | | | | | | | | | | |  |  |  |
| **S2d) Pitch** | | | | | | | | | | | | | | | | | | | | | | | |  |  |  |
|  | | | ***Pitch*** | | | | | | |  | | |  | |  | | | | | | |  | | |  |  |
|  | | | ***0.3*** | | ***0.5*** | ***0.8*** | | ***1.2*** | | ***r_s_*** | | | ***p*** | |  | | | | | | |  | | |  |  |
| ***Mean Error*** | | | 13.9 | | 14.8 | 10.0 | | 13.8 | | -0.60 | | | 0.417 | |  | | | | | | |  | | |  |  |
| ***Image Noise*** | | | 35.2 | | 35.3 | 38.2 | | 38.2 | | 1.0 | | | 0.083 | |  | | | | | | |  | | |  |  |
|  | | | | | | | | | | | | | | | | | | |  |  |  |  |  |  |  |  |
| **S2e) Reconstruction Kernel** | | | | | | | | | | | | | | | | | | |  |  |  |  |  |  |  |  |
|  | | ***Kernel*** | | | | | | | | | | | | | | | |  |  | | | |  |  |  |  |
|  | | ***Qr32*** | | ***Qr36*** | | ***Qr40*** | | ***Qr44*** | ***Qr49*** | | | ***Qr54*** | | ***Qr59*** | | ***Qr69*** | | ***r_s_*** | ***p*** | | | |  |  |  |  |
| ***Mean Error*** | | 15.4 | | 15.4 | | 15.6 | | 15.8 | 15.7 | | | 15.4 | | 20.4 | | 21.5 | | 0.83 | 0.015 | | | |  |  |  |  |
| ***Mean Error (Kernel Qr32 – Qr54 only - posthoc)*** | | 15.4 | | 15.4 | | 15.6 | | 15.8 | 15.7 | | | 15.4 | | - | | - | | 0.6 | 0.242 | | | |  |  |  |  |
| ***Image Noise*** | | 24.4 | | 29.3 | | 35.5 | | 43.6 | 52.7 | | | 67.0 | | 82.9 | | 125.6 | | 1.0 | <0.001 | | | |  |  |  |  |

Results of measurement of mean error and image noise in all experiments.

a) At 100 kV / Sn150 kV the lowest possible dosage was at a CTDI_vol_ of 1.5 mGy. b) At 80 kV / 140 kV the lowest possible dosage was at a CTDI_vol_ of 3 mGy; *CTDIvol* volume computed tomography dose index; *r_s_* Spearman rank correlation coefficient; *Sn* Indicates use of 0.6mm tin filter;
